# Encrypted immunity mediated by peptides

**DOI:** 10.1371/journal.pbio.3003914

**Published:** 2026-08-19

**Authors:** Zhenrun J. Zhang, Yifat Merbl, Cesar de la Fuente-Nunez

**Affiliations:** 1 Departments of Psychiatry and Microbiology, Perelman School of Medicine, University of Pennsylvania, Philadelphia, Pennsylvania, United States of America; 2 Institutes for Biomedical Informatics and for Translational Medicine and Therapeutics, Perelman School of Medicine, University of Pennsylvania, Philadelphia, Pennsylvania, United States of America; 3 Departments of Bioengineering and of Chemical and Biomolecular Engineering, School of Engineering and Applied Science, University of Pennsylvania, Philadelphia, Pennsylvania, United States of America; 4 Department of Chemistry, School of Arts and Sciences, University of Pennsylvania, Philadelphia, Pennsylvania, United States of America; 5 Penn Institute for Computational Science, University of Pennsylvania, Philadelphia, Pennsylvania, United States of America; 6 Department of Systems Immunology, Weizmann Institute of Science, Rehovot, Israel

## Abstract

Computational and experimental advances have led to the discovery of a large and diverse repertoire of bioactive peptides encrypted within proteins from across the tree of life. Mounting evidence suggests that these peptides can contribute to host defense not only through direct antimicrobial activity, but also through immunomodulatory and potentially broader physiological functions. This Essay synthesizes this emerging field from a broader biological perspective and explains the conceptual logic of immunity mediated by encrypted peptides. It proposes that such ‘encrypted immunity’ represents a previously underappreciated layer of host defense and physiology, with potential implications for therapeutics, diagnostics, and fundamental biology.

## Abstract 2

Encrypted peptides are short bioactive fragments hidden within larger proteins that remain largely undercharacterized, obscuring their roles in immunity. This Essay explores how proteolytically released peptides are discovered and how their mechanism of action helps to shape immunity and physiology.

## Introduction

Encrypted peptides are short amino-acid sequences (typically fewer than 50 residues) embedded within larger proteins [1]; in other words, they are fragments of natural proteins. Traditional methods for bioactive peptide discovery have led to the identification of a handful of encrypted peptides released from larger proteins, hormones, or pro-peptides. These include the thrombin-derived host-defence fragment GKY25 [2], and the human cathelicidin LL-37, which is liberated from its hCAP-18 precursor [3]. Other well-characterized encrypted peptides include lactoferricin B, which is generated by pepsin cleavage of bovine lactoferrin [4]; buforin II, a potent antimicrobial fragment of histone H2A found in stomach tissue from the Asian toad [5]; hemocidins derived from hemoglobin [6]; and the complement anaphylatoxin C3a, the processed form of which exhibits direct antibacterial activity [7]. Despite these discoveries, for a long time progress stagnated because hunting for novel encrypted peptides demanded painstaking fractionation, peptide sequencing, and bioactivity testing [8,9].

The landscape has now changed substantially. Algorithms have been developed that consider the amino-acid composition, sequence, and physicochemical properties of previously described antimicrobial peptides (AMPs) [10,11] and have led to encrypted peptides being discovered across the tree of life, including within the proteomes of humans [11–13], bacteria [14–16], archaea [17], and even extinct species [18,19]. Intriguingly, many antimicrobial encrypted peptides identified in the human proteome originate from non-immune proteins, yet exhibit immunomodulatory properties [1]. This observation led us to propose the ‘cross-talk hypothesis’, whereby non-immune proteins engage with the immune system through encrypted peptides, thereby contributing to host defense in ways not evident from their canonical annotation [1].

Over the past decade, rapid advances in machine learning (ML) and artificial intelligence (AI) have accelerated peptide discovery across biological systems. AI/ML models, including random forests, neural networks, and transformers, have facilitated the identification of novel antibiotics, including encrypted peptides, small-open-reading-frame-encoded peptides (SEPs), and archaic and modern encrypted peptides (AEPs and MEPs). Importantly, these tools have enabled systematic exploration across broad evolutionary scales, expanding our view of how widespread such peptides may be. However, although AI and ML have been instrumental in surfacing these hidden peptides at scale, the central questions now are biological rather than purely computational. When are these peptides generated? Which proteases or degradation pathways produce them? In which tissues or disease states are they most relevant? Are they regulated outputs of protein turnover, or merely stochastic byproducts of degradation? And beyond antibacterial activity, might encrypted peptides influence immune signaling, tissue homeostasis, metabolism, or neurobiology?

In this Essay, we discuss recent advances in encrypted peptide discovery while broadening the focus beyond AI/ML methods alone. We examine sources of encrypted peptide diversity in the human proteome, including proteolysis, proteoform (Box 1) variation, and tissue context, and discuss the biological logic of the cross-talk hypothesis and the emerging concept of ‘encrypted immunity’ (Box 1), including immunomodulatory and non-antibacterial functions of encryted peptides, placing these discoveries within a wider evolutionary framework across the tree of life. We also highlight key limitations, assumptions, and open questions that must be addressed if encrypted immunity is to mature from an appealing concept into a rigorous biological framework.

**Box 1. Glossary**

**Proteoform**

The specific molecular form of a protein arising from a given gene, including differences caused by alternative splicing, sequence variation, proteolytic processing, and post-translational modifications.

**Encrypted immunity**

A proposed layer of host defense in which bioactive peptides (or other functional biomolecules) hidden within larger proteins are released, typically through proteolysis, and contribute to antimicrobial activity, immune signaling, inflammation, or tissue homeostasis.

**Cardin and Weintraub motifs**

Heparin-binding sequence patterns enriched in basic residues, commonly represented as XBBBXXBX or XBBXBX, where B is a basic amino acid and X is hydrophobic or uncharged.

**ESKAPEE pathogens**

Highly drug-resistant bacteria: *Enterococcus faecium, Staphylococcus aureus, Klebsiella pneumoniae, Acinetobacter baumannii, Pseudomonas aeruginosa, Enterobacter spp*, and *Escherichia coli*.

**Retro-inverso variant**

A peptide analog composed of D-amino acids in reverse sequence order, designed to preserve the spatial orientation of side chains while improving resistance to proteolytic degradation.

**Immunosin**

A term used here for encrypted peptides derived from immune-related proteins.

**Random forests**

An ensemble machine-learning method that combines many decision trees to generate a more robust classification or regression prediction.

**Molecular de-extinction**

The resurrection of molecules of life from ancient biology.

## Encrypted peptide discovery in the human proteome

Human proteins are constantly remodeled by proteolysis. More than 500 human proteases, distributed across five catalytic classes [20], are encoded in the genome and have a broad spectrum of specificities, ranging from relatively nonspecific hydrolysis to highly selective cleavage of defined substrates. In parallel, proteasomes, lysosomes, and autophagy-associated pathways contribute to protein turnover in highly compartmentalized and regulated ways. Together, these systems create an enormous and dynamic peptide output from the human proteome.

The diversity of encrypted peptides is shaped not only by protease specificity, but also by the tightly regulated proteolytic processes operating in distinct cellular compartments such as proteasomes, lysosomes, and autophagosomes. Indeed, many encrypted peptides are predicted to be generated via proteolysis of larger proteins [18], including in autophagosomes, where peptides generated from cytosolic proteins were found that exert antimicrobial activity against *Mycobacterium tuberculosis* [21]. Bioactive peptides that are ‘hidden’ or ‘encrypted’ within larger precursor proteins and released through biological processing to perform distinct functions, have previously been described as cryptides and noted to be prevalent across the animal and plant kingdoms [22].

The sequence diversity of encrypted peptides is further enhanced by proteoform diversity. Although the human genome contains approximately 20,000 protein-coding genes, alternative splicing events, wherein precursor messenger RNA sequences are arranged in various combinations, significantly expand the proteoform repertoire. RNA sequencing studies of human organs have estimated that transcripts from more than 95% of multi-exon genes undergo alternative splicing [23,24]. Recent single-cell transcriptome sequencing studies have further revealed that true splice isoform complexity may exceed previous estimates [25,26]. Additional sources of proteome variation, such as single-amino acid polymorphisms (SAPs) and post-translational modifications, may further amplify the complexity of the human proteome [27–30], and hence the diversity of potential encrypted peptides. Moreover, it remains to be elucidated whether certain regulatory mechanisms are at play to drive the generation of encrypted peptides under different conditions, or whether these peptides are merely samples from the expressed repertoire.

## Discoveries pre-computational screening

**Fig 1:**
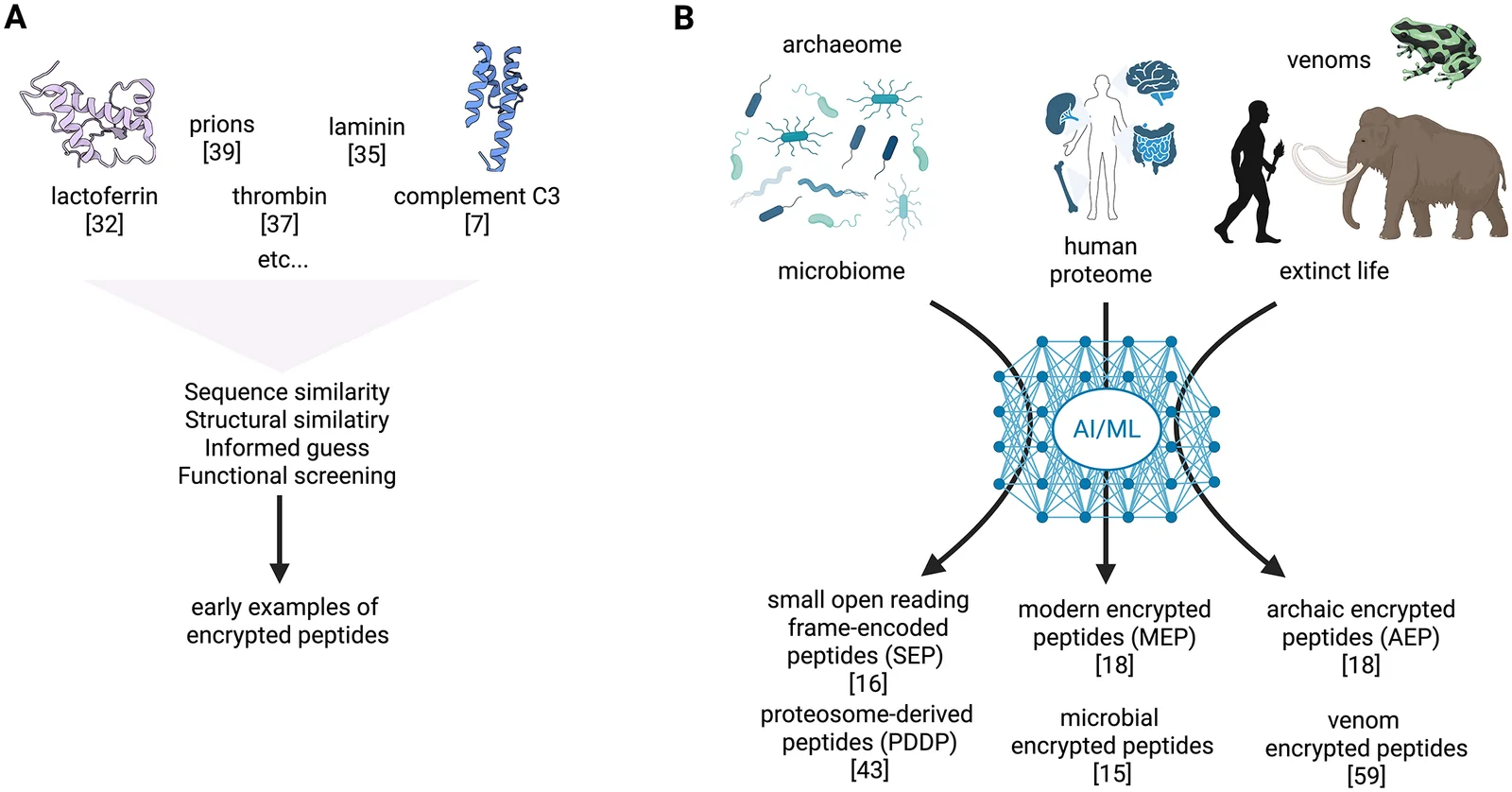
Discovery of encrypted peptides. **A.** Early discovery of encrypted peptides in human proteins with sequence similarity from informed guesswork, verified by biochemical analyses, yielded a few examples including peptides derived from lactoferrin [32], thrombin [33], complement C3 [7], prions [34], and laminin [35]. **B.** Computational approaches have been used to uncover numerous bioactive petides across the tree of life, including in living and extinct species. These include proteasome-derived defense peptides (PDDP) [36], peptides from modern humans (MEP) and from archaic human ancestors (AEP) [18], peptides from small open-reading frames (SEP) [16], peptides from microbes [15], and peptides from venoms [37]. Created in BioRender. https://BioRender.com/w5ui680.

Long before proteome-wide computational screens, biochemical studies hinted that human proteins could harbor latent antimicrobial fragments (Fig 1A). For example, lactoferrin, an iron-binding glycoprotein found in mammalian biological fluids including milk and mucous secretions, was initially recognized for its antimicrobial properties, which were attributed to environmental iron sequestration [31]. However, peptides derived from human and bovine lactoferrins through pepsin cleavage exhibited bactericidal properties that were more potent than the intact protein [32]. The active peptide consisted of residues 1–47 at the N-terminus of human lactoferrin, an area that contains intramolecular disulfide bonds, and this peptide was highly active against Gram-positive and gram-negative pathogens, with a minimal inhibitory concentration (MIC) in the range of 0.3 to 3 mM [32].

A second example involves heparin-binding peptides. Building on the observation that human antimicrobial peptides LL-37 and α-defensin bind to glycosaminoglycans [38,39], Andersson *and colleagues* hypothesized that peptides with heparin-binding motifs might possess antimicrobial activity. The heparin-binding peptide sequences (Cardin and Weintraub motifs; Box 1) follow the pattern XBBBXXBX or XBBXBX, where X represents hydrophobic or uncharged amino acids, and B represents basic amino acids. Indeed, peptides containing these motifs from laminin, fibronectin, von Willebrand factor, complement factor C3, and other proteins exhibit antimicrobial activity against gram-negative and Gram-positive bacteria, as well as against *Candida albicans* [35]. Similarly, the histidine-rich region of histidine-rich glycoprotein contains the peptide (GHHPH)_4_, which is effective against *Enterococcus faecalis* [40].

Another notable example is thrombin, a key enzyme in the coagulation cascade. Digestion of human thrombin by neutrophil elastase yields peptides with antimibrobial activity, whereas the intact protein is inactive [33]. In particular, thrombin-derived C-terminal peptides (TCPs) showed strong antimicrobial activities against multiple pathogens. Importantly, TCPs were detected in samples from human ulcers and at bacterial surfaces in vivo in fibrin from human wounds, suggesting their physiological significance in human wounds. Other human proteins containing encrypted peptides that were discovered prior to the introduction of large-scale computational approaches include complement C3 [7], kininogen [41], prions [34], β2-glycoprotein [42], and tissue factor pathway inhibitor [43] (Fig 1A).

## Discoveries in the age of computational screening

Systematic human proteome mining has since expanded this principle considerably. One approach is to apply a scoring function to identify antimicrobial sequences within peptides; for example, a scoring function can evaluate the physicochemical properties of peptides, such as net charge, average hydrophobicity, and sequence length, all of which can then be integrated into a fitness function [44]. By using this framework to screen peptides, 2,603 putative antimicrobial encryted peptides were discovered that fell into two main categories: previously undescribed encryted peptides, and peptide hormones with putative functions unrelated to antimicrobial properties [11]. Fifty-five encryted peptides were subsequently synthesized, the majority of which (63.6%) exhibited antimicrobial activity against at least one ESKAPE pathogen (Box 1). Notably, some peptides targeted human gut and skin commensals, suggesting their potential roles in regulating microbiota on mucosal surfaces. Interestingly, encrypted peptides from signal peptide, CUB and EGF-like domain-containing protein 1 (SCUBE1) and SCUBE3 showed potent activity against *Acinetobacter baumannii* individually and also exhibited synergy when tested together [11]. These two proteins are expressed in endothelial tissues, suggesting their potential involvement in innate defense. Similarly, among 39 encrypted peptides derived from different tissues of the human body, 36 exhibited activities against at least one ESKAPE pathogen or commensal bacteria, with several encrypted peptide pairs from the same tissue or organ context demonstrating synergistic effects (e.g., collagenins 3 and 4 from collagen α1(XVI) chain against *A. baumannii*) [1]. In another study, peptides from human apolipoprotein B displayed robust activity against several pathogens in vitro and in vivo, and a retro-inverso variant (Box 1) of the lead encrypted peptide enhanced its proteolysis resistance in serum, improving its anti-infective efficacy in a *Pseudomonas aeruginosa* skin infection model [13].

A previously underappreciated source of human encrypted peptides has emerged from proteasome-mediated protein degradation. A fundamental role for proteasome-derived defense peptides (PDDPs) was recently uncovered in innate immunity [36]. A comprehensive proteome-wide computational analysis identified hundreds of thousands of putative PDDPs, suggesting a vast and untapped repertoire of bioactive peptides within the human proteome [36]. Interestingly, PDDP-containing proteins have diverse tissue specificities, suggesting that PDDPs may also exhibit distinct patterns across tissues. Such PDDPs can be detected through mass spectrometry analysis of proteasome-cleaved peptides [45,46], which are constitutively generated during protein degradation and exhibit antimicrobial activity by disrupting bacterial membranes. Notably, PDDPs generated from the full-length proteins PPP1CB and PSMG2 can kill intracellular *Salmonella typhimurium*, and PPP1CB-derived peptide reduced the bacterial burden in bacteremia and pneumonia infection models with *P. aeruginosa* [36]. The production of PDDPs is enhanced upon bacterial infection via alterations in proteasomal composition and function, particularly through the recruitment of the PSME3 regulatory subunit, which shifts cleavage specificity towards tryptic-like cleavages [36]. Similarly, the deep learning model APEX has been used to identify proteasome-derived peptides with potential antibiotic properties [47].

These findings expand our understanding of proteasome function beyond antigen processing and presentation, emphasizing its direct role in host defense and highlighting proteolytic-driven immunity by both the adaptive and innate arms of the immune system. Beyond their antimicrobial activity, PDDPs may have additional functions as context-sensitive immune modulators. Yet, whether their production is regulated by cellular stress and immune cues remains to be elucidated. It is tantalizing to speculate that proteolytically generated peptides, collectively termed the ‘degradome’, may act as a flexible output system tuned by tissue type, pathogen class, or stress state.

Looking across species, the diversity of encrypted peptides has been explored in modern humans and in extinct human ancestors using a protease-oriented approach [18]. The panCleave random forest model was developed for proteome-wide cleavage site prediction, yielding MEP fragments from modern humans and AEP fragments from Denisovans and Neanderthals, some of which demonstrated anti-infective efficacy against *A. baumannii* in mouse models of infection. Taken together, these recent computational screens suggest that many more encrypted peptides and PDDPs may still remain to be discovered. The explosion in *in silico* discovery, spanning millions of candidate peptides, underscores a growing need for functional filters that go beyond sequence features. Without biological context, including tissue of origin, proteolytic route, stress response linkage, and in vivo regulation, we risk overestimating the physiological significance of these sequences.

## Biology of human encrypted peptides

Encrypted peptides derived from the human proteome (and AEPs), exhibit unique physicochemical properties, diverse mechanisms of action for their antimicrobial activity, and multimodal effects on host cells beyond their antimicrobial functions. Previously identified AMPs, as cataloged in public databases such as Database of Antimicrobial Activity and Structure of Peptides (DBAASP), often contain motifs and repeats of cationic and hydrophobic residues, forming amphipathic structures that are critical to their mechanisms of action. By contrast, human encrypted peptides that were discovered using the previously mentioned scoring function [11] contained a higher percentage of hydrophobic (16.3%) and basic (8.1%) residues, along with lower proportions of polar (5.8%) and acidic (68.0%) residues. These findings suggest that human encrypted peptides may constitute a novel class of natural AMPs that do not necessarily rely on amphipathic structures but instead feature arginine-rich, slightly hydrophobic sequences. Moreover, AEPs are characterized by lower amphiphilicity, a higher propensity for disorder, and reduced aggregation potential [19], suggesting that APEX-identified AEPs represent a distinct family of AMPs with increased uncharged polar residues and higher aliphatic content. However, caution should be noted as different algorithms may yield distinct and variable predictions, and the rate of experimental validation is still lagging behind.

These differences in physicochemical properties may translate to variations in the structures and mechanisms of action of encrypted peptides. Traditional AMPs often adopt α-helical structures; interestingly, the human encrypted peptides discovered using the scoring function have a greater prevalence of anti-parallel β-strands and are predominantly unstructured, even in helix-inducing media [1]. By contrast, AEPs and MEPs identified by APEX show predominantly helical structures, despite their unusual abundance of uncharged polar residues and low amphiphilicity [19]. Regarding the mechanism of action, AMPs typically interact nonspecifically with the lipid bilayer of bacteria, with outer membrane permeabilization being the most common mechanism. Indeed, the MEPs that have been tested are effective at permeabilizing the outer membrane but not at disrupting the cytoplasmic membrane of *A. baumannii* [11]. Conversely, the AEPs that have been tested demonstrated superior depolarization of the cytoplasmic membrane, surpassing both polymyxin B and MEPs, despite being less effective at outer membrane permeabilization [19]. However, the drawback of their unspecific mechanism of action is cytotoxicity. Certain encrypted peptides exhibit cytotoxicity at concentrations similar to their MICs against pathogens, limiting their therapeutic potential; however, other encrypted peptides have demonstrated no notable cytotoxicity at tested concentration well above their MICs, indicating a broad therapeutic window and making them promising candidates for therapeutic development.

**Fig 2:**
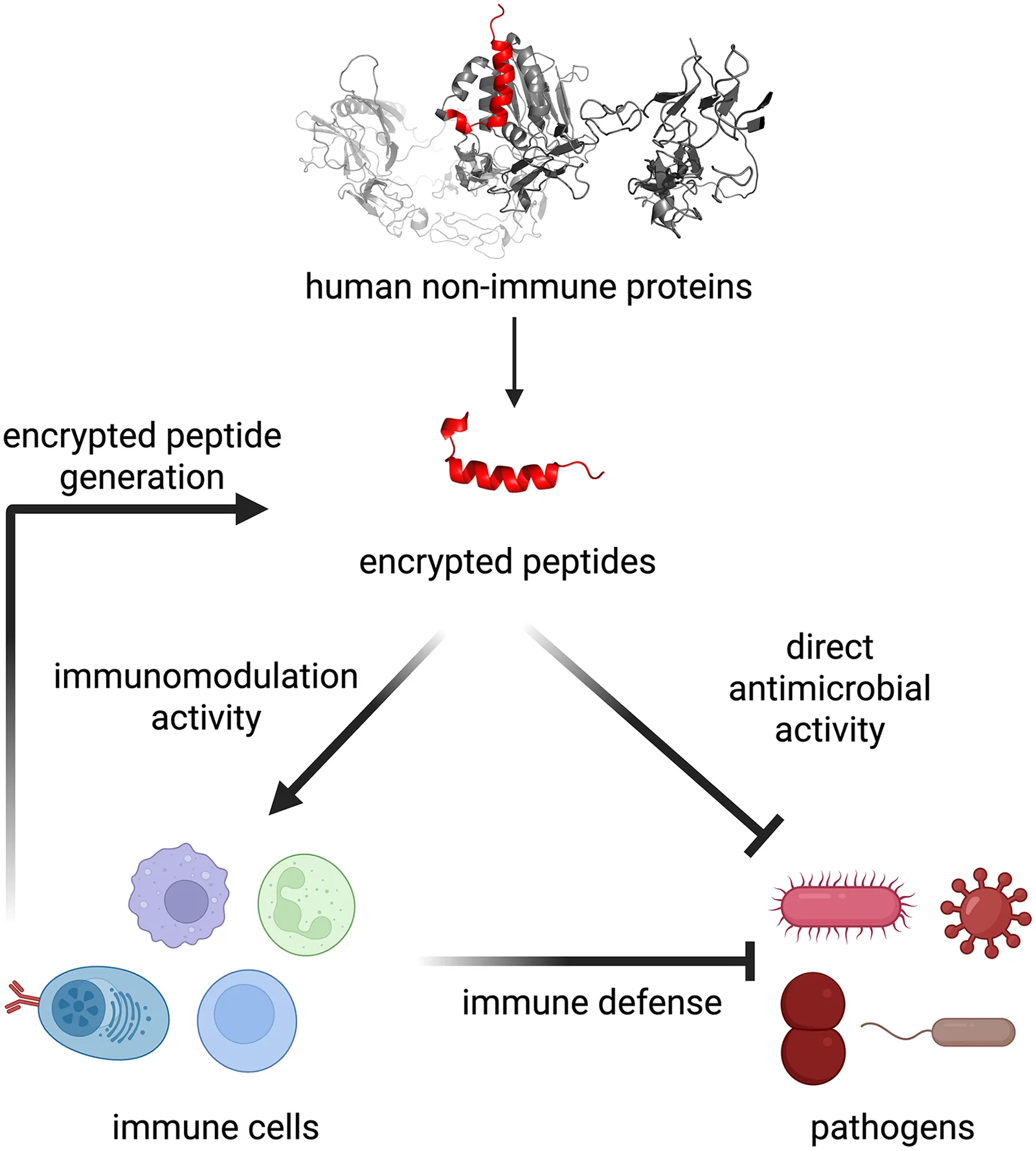
Conceptual model of encrypted immunity. Proteins expressed in immune and non-immune tissues can be processed by proteases, the proteasome, or autophagy-associated pathways to release encrypted peptides. Depending on the tissue context and biological state, these peptides may exert direct antimicrobial effects, modulate inflammatory signaling, alter chemotaxis, or participate in broader physiological processes. Examples informing this model include human proteome-derived encrypted peptides, thrombin-derived C-terminal peptides, and proteasome-derived defense peptides. Infection, injury, or inflammatory cues may in turn reshape peptide generation, creating a dynamic feedback system between proteolysis and host defense. Created in BioRender. https://BioRender.com/06cyf96.

Interestingly, apart from their antimicrobial activities, some human encrypted peptides also exhibit immunomodulatory effects on host cells. For example, collagenin-2, derived from the metalloproteinase ADAM12, is anti-inflammatory, reducing the amount of pro-inflammatory cytokines IL-6 and MCP-1 produced by human keratinocyte HaCaT cells, whereas collagenin-5, derived from collagen α6(VI) chain (CO6A6), is pro-inflammatory [1]. Similarly, some immunosins (Box 1) increase the amounts of the pro-inflammatory cytokines TNF, IL-6, and MCP-1 in PMA-differentiated human monocyte THP-1 cells, whereas other immunosins display anti-inflammatory effects. Notably, 68% of tested encrypted peptides cause an increase in MCP-1, a chemoattractant that recruits diverse immune cells [1]. Additionally, TCPs from thrombin inhibit the release of nitric oxide and TNF from LPS-stimulated macrophages, and significantly reduce pro-inflammatory cytokine production and improve survival in an LPS-induced mouse model of septic shock [33]. These findings support the ‘cross-talk hypothesis’, wherein non-immune proteins engage with the immune system via peptides, contributing to their overall function (Fig 2). Additional potential effects of encrypted peptides on host cells and physiology warrant further investigation.

Although the mechanisms of action of the immunomodulatory effects of human encrypted peptides have not been explored, parallels may be drawn from well-characterized human AMPs [48]. For example, LL-37 can modulate innate immune responses by interacting with Toll-like receptors to suppress pro-inflammatory cytokine secretion by monocytes [49]. Conversely, the human α-defensin HNP1-3 increases the production of pro-inflammatory cytokines in monocytes [50]. Human α-defensins also act as chemokines to recruit innate immune cells, whereas β-defensins achieve similar functions through interaction with the chemokine receptor CCR2 [51]. Interestingly, some synthetic AMPs have also shown a similar chemoattractant function [52,53]. In adaptive immunity, human β-defensins and LL-37 selectively interact with chemokine receptor CCR6 to recruit immature dendritic cells (DCs) to infection sites [51]. LL-37 also enhances the secretion of T helper 1 cell-inducing cytokines by DCs and promotes T helper 1 cell responses [54]. Moreover, defensins may act as adjuvants, boosting specific Immunoglobulin G antibody production by B cells [55]. In fact, some AMPs exert their effects primarily through immunomodulation rather than through direct pathogen killing; for instance, the frog-derived cathelicidin PopuCATH, while lacking in vitro antibacterial activity, effectively reduces bacterial burden and inflammation in vivo [56]. We speculate that novel human encrypted peptides possess similar multifunctional characteristics and may employ comparable strategies in vivo for infection control (Fig 2).

## Discovery of encrypted antimicrobial peptides across the tree of life

Advances in high-throughput DNA sequencing and large-scale genomics and metagenomics have enabled the exploration of genomes and metagenomes from diverse organisms, environments, and even from extinct species. The wealth of genomic and metagenomic sequences provides a valuable resource for the discovery of novel peptide antibiotics through bioinformatic mining.

Recent computational advances, particularly in AI and ML, have revolutionized the discovery of antibiotics in bacteria and archaea. For example, Macrel is a pipeline that uses random forests (Box 1) to predict antibiotic peptides from large peptide datasets, prioritizing precision over recall [57]. This tool was applied to globally distributed public metagenomes and high-quality bacterial and archaeal genomes, resulting in the AMPSphere collection, which comprises 863,498 non-redundant candidate AMP sequences [15]. Notably, out of 100 synthesized peptides from AMPSphere, 79 demonstrated antimicrobial activity, and 63 were effective against ESKAPE pathogens in vitro. Similarly, AmPEP is another random forest classifier designed to predict antimicrobial potential in query sequences that can be used to screen for AMP candidates within small open-reading frames (smORFs) in human microbiome metagenomes [58]. This approach revealed 323 candidate SEPs, of which 78 were synthesized and tested, and 55 exhibited antimicrobial activity [16]. Interestingly, lead candidates, such as prevotellin-2 from a gut-associated *Prevotella copri* strain, displayed potent activity against pathogens while sparing commensal bacteria.

Natural language processing models can autonomously learn sequence features and have also been applied to AMP identification, even for short sequences with low homology to known AMPs. One approach is to combine multiple neural network architectures, including long short-term memory, attention mechanisms, and bidirectional encoder representations from transformers, to identify key antimicrobial sequence features [14]. The optimized model produced using this approach achieved high precision and recall in AMP prediction and was subsequently applied to smORFs from human gut metagenomes, identifying 2,349 candidate AMPs. Of the 216 chemically synthesized peptides, 181 (>83%) showed antimicrobial activity. Several top candidates also demonstrated efficacy in a mouse model of *Klebsiella pneumoniae* lung infection. A different approach is to use a deep learning model such as APEX, which uses a multitask learning architecture to predict the antimicrobial activity of a given amino acid sequence [19]. It utilizes an encoder neural network that combines recurrent and attention neural networks to extract hidden features from peptide sequences. APEX has been used to explore extinct organisms for molecular de-extinction (Box 1) of peptide antibiotics, which resulted in the discovery of novel compounds that depolarize the cytoplasmic membrane of bacteria, distinct from traditional outer membrane-targeting AMPs [19]. Similar deep learning approaches have also been applied to discover novel peptides with antimicrobial activity in the archaeome [17], among proteasome-derived peptides [47], and in venoms [37] (Fig 1B). Models such as APEX promise to unlock even deeper insights into the hidden repertoire of encrypted peptides and other peptides, both within the human proteome and beyond.

## Limitations, assumptions, and open questions

A more critical framing is now essential for the field. First, not every peptide released during protein turnover is likely to be functional—that is, capable of exerting direct antimicrobial, immunomodulatory, signaling, or other measurable physiological effects. The existence of a peptide sequence with favorable physicochemical features does not guarantee that it is generated at meaningful abundance in vivo, persists long enough to act, reaches the right compartment, or contributes measurably to physiology. Distinguishing functional encrypted peptides from inert degradation byproducts remains a central challenge.

Second, the reliable detection of human encrypted peptides from clinical samples poses a significant challenge. Although computational models predict their presence and potential roles in the body, direct experimental validation through patient-derived specimens has been limited. Proteomics workflows, which typically fragment proteins for liquid chromatography-tandem mass spectrometry analysis (bottom-up proteomics), make it challenging to discriminate intact encrypted peptides from other peptides generated during sample processing. This process obscures the reliable detection of intact bona fide encrypted peptides, as they become indistinguishable from other peptide fragments generated in vitro. Improved methodologies are needed to identify bona fide encrypted peptides in biological samples reliably. More robust approaches will likely require dedicated peptidomics, activity-guided fractionation, targeted mass spectrometry, spatially resolved sampling, and orthogonal biochemical validation in native tissues and clinical samples. Early examples of methods to detect circulating human peptides from human hemofiltrate [59] suggest a promising direction for encrypted peptide detection in the future.

Third, regulation is poorly understood. For most encrypted peptides, we still do not know the answer to several key questions, including: which proteases generate them under physiological conditions; how their release is controlled; how rapidly they are degraded; whether they are modified after release; or how infection, stress, cytokines, or tissue injury alter their production. The degradome is dynamic, but the logic governing that dynamism remains largely uncharted. In addition, the functional readouts available are still too narrow. Much of the research in this field has been driven by antimicrobial screening, which was a sensible starting point given the urgent need for antibiotics. But many encrypted peptides could act primarily as immune modulators, ecological regulators, metabolic signals, or context-dependent cofactors rather than as direct antibiotics. Assays that focus only on MIC will miss much of this biology.

Fourth, computational discovery carries its own assumptions. Models trained on known AMPs or related datasets may enrich for peptides that resemble previously characterized classes, thereby under-sampling genuinely novel functional space. Different models can prioritize different physicochemical regimes, but experimental validation still lags far behind *in silico* prediction. As candidate counts expand from thousands to millions, biological triage will become at least as important as computational scoring.

Finally, the evolutionary interpretation of encrypted peptides is not straightforward. Some may be under direct selection, others may arise opportunistically from precursor sequences shaped for entirely different reasons. Demonstrating evolutionary conservation, regulated release, or recurrent functional reuse across contexts will be important for distinguishing adaptive signals from incidental chemistry.

## Conclusions and future perspectives

The emerging field of encrypted immunity, mediated by peptides and other functional biomolecules, suggests that host defense may be distributed more broadly across the proteome than previously appreciated. Proteins not classically assigned to roles in immunity may nonetheless contribute to defense, inflammation, and tissue homeostasis through bioactive fragments that are released in a context-dependent manner. Within this view, the proteome contains not only canonical functional domains, but also a hidden layer of conditional effector potential.

Recent work has made this hidden layer visible, revealing encrypted peptides and related peptides across human tissues, microbiomes, archaea, venoms, and extinct organisms. Yet the next phase of research for the field should move beyond discovery alone. The pressing challenge is to define the regulatory logic that governs peptide generation, fate, and function: which precursor proteins are mobilized in which tissues, by which proteolytic routes, under which biological conditions, and toward which outcomes. Integrating protease specificity, tissue expression, pathogen exposure, stress responses, and peptide stability will be essential for separating physiologically relevant effectors from the background of protein turnover.

If this framework holds, it could have important translational implications. Endogenous encrypted peptides with favorable activity and therapeutic windows may provide starting points for antimicrobial or immunomodulatory therapy development. Proteolytic pathways that release such peptides might themselves become targets for therapeutic manipulation. Encrypted peptides and related peptide fragments might also serve as biomarkers of infection, inflammation, immune status, or tissue injury. Encrypted peptides could also have metabolic and neuroactive functions beyond immunity, exemplified by the recently identified BRINP2-related peptide (BRP), which has been shown to reduce food intake and exert anti-obesity effects and originates from prohormone convertase-mediated cleavage of BRINP2, a protein with no prior association with appetite regulation [60]. At the same time, recent success stories involving engineered peptide therapeutics, including the GLP-1 receptor agonists liraglutide [61] and semaglutide [62] (derived from the endogenous peptide hormone GLP-1 [63]) and the dual GLP-1 receptor/GIP receptor agonist tirzepatide [64] (derived from both GLP-1 and peptide hormone GIP), illustrate the broader power of translating latent biological signals into medicines through interdisciplinary collaboration. Recent work on transthyretin further supports this principle: it identified this classical thyroid hormone and retinol transporter as an innate immune effector against gram-negative bacteria and mapped the activity to an N-terminal encrypted peptide that disrupts bacterial membrane integrity [65].

Advances in AI/ML algorithms and computational power, combined with large-scale sequencing of life on Earth, should continue to reveal hidden repertoires of encrypted peptides and other encrypted biomolecules across biological kingdoms [15,16]. Beyond extant life, sequencing archaic animal and human samples, paired with molecular de-extinction technologies [18,19], may help unravel how these peptides have been conserved or adapted over evolutionary time. The resulting knowledge could guide new strategies for peptide engineering, ultimately expanding the scope and impact of peptide-based therapeutics. More broadly, beyond the encrypted peptides discussed in this Essay, additional functional biomolecules, including oligonucleotides, microproteins, glycans, lipids, metabolites, and combinatorial conjugates, likely remain to be uncovered and may prove important in host immunity, physiology, and other fundamental processes of life.

## Abbreviations

AEP: archaic encrypted peptide
AI: artificial intelligence
AMP: antimicrobial peptide
BRP: BRINP2-related peptide
DC: dendritic cell
MEP: modern encrypted peptide
MIC: minimal inhibitory concentration
ML: machine learning
PDDP: proteasome-derived defense peptide
SAP: single-amino acid polymorphism
SEP: small-open-reading-frame-encoded peptide
smORF: small open-reading frame
TCP: C-terminal peptide
